# Reduced composite dietary antioxidant index increases the risk of Parkinson’s disease and all-cause mortality in Parkinson’s disease patients: evidence from the NHANES database

**DOI:** 10.3389/fnagi.2025.1510654

**Published:** 2025-04-17

**Authors:** Fei Huang, Jingwen Hao, Chanjuan Chen, Qun Liu, Dan He

**Affiliations:** Department of Neurology, The First Hospital of Changsha, Changsha, China

**Keywords:** Parkinson’s disease, NHANES database, composite dietary antioxidant index, all-cause mortality, restricted cubic spline regression model

## Abstract

**Background:**

This study aimed to investigate the relationship between the Composite Dietary Antioxidant Index (CDAI) and the prevalence of Parkinson’s disease (PD), as well as to explore its relationship with all-cause mortality risk in PD patients.

**Methods:**

Data from the National Health and Nutrition Examination Survey (NHANES) database spanning from 2007 to 2018 were used, including 119,609 participants. After excluding individuals aged <18 years, those with incomplete follow-up data, and those missing critical variables such as CDAI and covariates, the final cohort consisted of 34,133 participants. Participants were categorized into a PD group (510 individuals) and a non-PD group (33,623 individuals). The CDAI values were calculated, and participants were divided into three groups based on the tertile distribution of their CDAI scores: Q1 (CDAI < −1.07), Q2 (−1.07 to 1.74), and Q3 (CDAI >1.74). Weighted logistic regression and weighted Cox regression analyses were employed to evaluate the associations between CDAI and the prevalence of PD, as well as between CDAI and all-cause mortality risk. Restricted cubic spline regression analysis was used to further elucidate the precise relationship between CDAI and outcome events.

**Results:**

CDAI values were significantly lower in the PD group compared to the non-PD group. After adjusting for age, sex, comorbid conditions (hypertension and diabetes), blood lipid and glucose levels, a reduction in CDAI was associated with an increased risk of PD (Q3 vs. Q1, OR = 0.72, *p* = 0.035). In patients with PD, a decrease in CDAI was significantly associated with a higher risk of all-cause mortality (Q3 vs. Q1, HR = 0.53, *p* = 0.018). This association was particularly pronounced in those over 60 years old, smokers, and those with hypertension. Restricted cubic spline regression analysis identified CDAI <0.471 as a risk factor for PD, and CDAI <0.527 as a risk factor for all-cause mortality in PD patients.

**Conclusion:**

CDAI reduction is an independent risk factor for both PD risk in the general population and all-cause mortality in PD patients, with amplified predictive power in older adults, smokers, and hypertensive individuals. Our findings support developing personalized antioxidant-enhancing nutritional interventions for both high-risk populations with suboptimal CDAI and established PD patients.

## 1 Introduction

Parkinson’s disease (PD), the second most frequent neurodegenerative disorder after Alzheimer’s disease, has experienced a significant rise in global prevalence over recent decades (Zhu et al., 2024). Epidemiological data reveals an alarming 273.76% surge in PD cases between 1990 and 2021, escalating from 3.15 to 11.77 million cases globally, further projection models forecast a 1.5-fold increase in PD prevalence by 2035, potentially affecting over 17.27 million individuals worldwide (Luo Y. et al., 2024). PD presents a broad spectrum of symptoms, encompassing both motor symptoms such as tremors, rigidity, bradykinesia and non-motor symptoms such as cognitive impairment, depression and sleep disorders (Bloem et al., 2021). At present, no therapy is available that can halt the progression of PD. The progressive deterioration of PD symptoms, coupled with the side effects of treatment drugs, significantly impact the quality of life for individuals with PD, imposing substantial stress and socioeconomic burdens on both families and society. The annual economic burden of PD in the United States alone exceeds $51.9 billion in 2017, further projected total economic burden surpassing $79 billion by 2037 (Yang et al., 2020). Therefore, interventions to reduce PD incidence, delay disease progression, and alleviate symptom burden are of vital importance.

The pathological characteristic of PD is progressive loss of dopamine-producing neurons in the substantia nigra pars compacta (SNc) of midbrain, accompanied by the aggregation of *α*-synuclein and the formation of Lewy bodies in the remaining dopaminergic neurons (Zeng et al., 2018). Recent studies have identified oxidative stress as a critical role in the pathogenesis of PD (Salaramoli et al., 2023), contributing to neuronal death through various mechanisms, including mitochondrial dysfunction, dopamine metabolism, neuroinflammation, and impaired antioxidant defenses. Diet and oxidative stress are closely interconnected; daily diets can be categorized into antioxidant-rich diets (e.g., fruits, vegetables, nuts, seeds, whole grains) and pro-oxidative diets (e.g., processed foods, red meats, sugar) based on their effects on oxidative stress. Dietary patterns with a high intake of fruit, vegetables, legumes, whole grains, nuts, fish, and poultry and a low intake of saturated fat were inversely associated with PD risk (Gao et al., 2007; Chromiec et al., 2021; Zhang et al., 2022; Xu et al., 2023), whereas higher consumption of large amounts of saturated fat has been linked to an increased risk of PD (Hantikainen et al., 2022; Xu et al., 2023). However, some other studies have not clearly established a role for dietetic consumption of prooxidants and antioxidant in PD risk (Jimenez-Jimenez et al., 1999; Erro et al., 2018). The discrepancies among these studies stem from variations in methods for calculating dietary oxidative/antioxidant capacity and differences in the populations studied. Therefore, new methodologies for calculating dietary antioxidant capacity, along with large-scale population follow-up studies, are necessary to further elucidate the role of antioxidant-rich foods in PD.

The Comprehensive Dietary Antioxidant Index (CDAI) is a validated composite tool designed to assess the overall antioxidant capacity of an individual’s diet. Unlike simpler measures that evaluate only a few specific antioxidants or foods, the CDAI incorporates six dietary antioxidants-vitamins A, C, and E, carotenoids, selenium, and zinc-providing a more comprehensive assessment of dietary antioxidant intake (Zhou T. et al., 2024). Previous studies have shown that CDAI is associated with various health outcomes including olfactory dysfunction (Zhou T. et al., 2024), diabetic retinopathy (Qiao et al., 2024), carotid intima-media thickness in coronary heart disease (Rivas-Garcia et al., 2024), and colorectal cancer (Yu et al., 2022).

However, no studies have examined the relationship between CDAI and the risk of PD. Consequently, this study investigates the association between CDAI and the risk of PD and all-cause mortality in PD patients, as well as the influence of different population characteristics on the all-cause mortality in PD patients in the 2007–2018 NHANES database. We aim to provide a nutritional basis for the prevention and intervention of PD.

## 2 Materials and methods

### 2.1 Study population design

**Figure 1:**
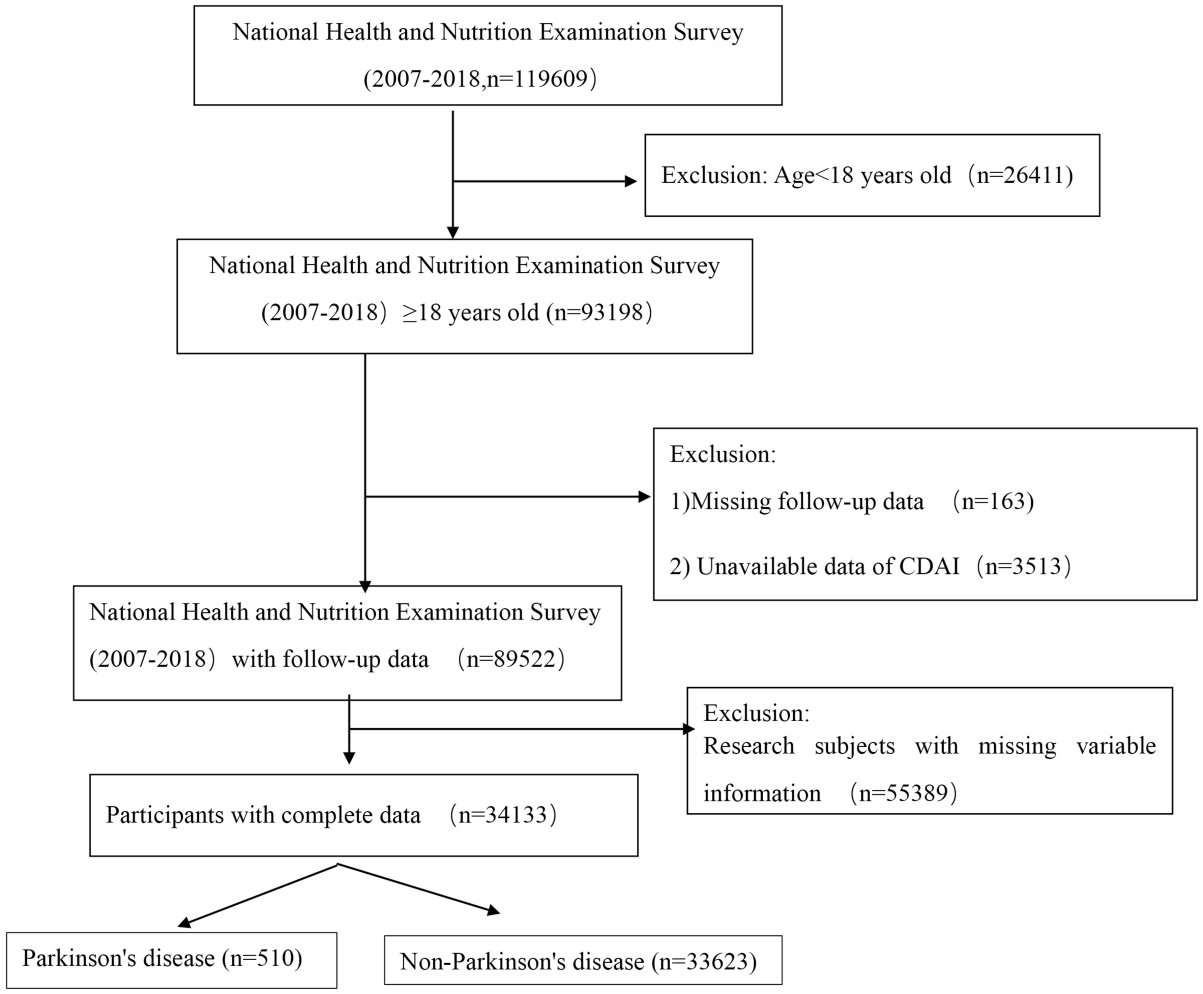
Flowchart of the participants’ selection from NHANES 2007–2018.

NHANES, an ongoing nationwide study, is designed to assess the dietary habits and physical health of adults and children across the United States. This study utilized data from 119,609 U.S. respondents obtained from the NHANES database^1^ spanning the years 2007 to 2018. Initially, 26,411 participants under the age of 18 were excluded due to their lack of follow-up data and incomplete information on several critical variables. Subsequently, 163 individuals without survival follow-up data and 3,513 individuals lacking core study outcome data for CDAI were excluded. Additionally, 55,389 individuals with incomplete covariate information were removed from the analysis. Finally, based on the screening criteria for PD, the remaining participants were categorized into two groups: 33,623 non-PD participants and 510 PD patients. A visual representation of the participant selection process is provided in Figure 1.

### 2.2 Exposure variable: comprehensive dietary antioxidant index (CDAI)

During the NHANES survey follow-up, participants’ intake of various nutrients and energy was comprehensively analyzed using two 24-h dietary recalls. Additionally, information on dietary supplements consumed during the same 24-h periods was also collected.

In this study, the raw measurement for each nutrient was obtained by summing two 24-h dietary intakes and dietary supplements. The averages of the two 24-h dietary intakes and the average of the two 24-h dietary supplement intakes were calculated separately, and then combined to obtain the measurement. It is important to note that, in the NHANES database (2007–2018), information on vitamin A and vitamin E from dietary supplements was not recorded. Therefore, raw measurements for vitamin A and vitamin E were based solely on dietary intake. For the remaining four nutrients, the raw measurements were calculated by the sum of dietary intake and additional dietary supplement intake.

The CDAI was calculated as according to the method described by Zhou T. et al. (2024), generally, standardized values for six dietary antioxidants (vitamins A, C, and E, carotenoids, selenium, and zinc) were derived by subtracting the mean and dividing by the standard deviation. The final CDAI score was then obtained by summing these standardized values (Zhou T. et al., 2024).


$$\mathrm{CDAI}=\overset{6}{\underset{i=1}{\sum }}\frac{\mathrm{Xi}-\mu i}{Si}$$

In this formula, Xi represents the raw measurement for each nutrient, μi denotes the mean of the raw measurements for all participants for that nutrient, and Si represents the standard deviation of the raw measurements for all participants for that nutrient.

### 2.3 Covariates

Age, gender, race, education level, and the ratio of family income to poverty (PIR) were obtained from demographic data, whereas body mass index (BMI), systolic blood pressure, and diastolic blood pressure were derived from physical examination measurements. Smoking history, alcohol consumption, history of hypertension and diabetes, as well as medication use, were all obtained from questionnaire data. Serum albumin, serum creatinine, alanine transaminase (ALT), aspartate transaminase (AST), triglycerides (TG), total cholesterol (TC), glycohemoglobin (GHB), and fasting blood glucose were measured according to standardized procedures.

Hypertension was defined by meeting any of the following criteria (Georgianos and Agarwal, 2016): (a) self-reported diagnosis of hypertension by a physician; (b) current use antihypertensive medication; or (c) three consecutive measurements of systolic blood pressure (SBP) > 140 mmHg and/or diastolic blood pressure (DBP) > 90 mmHg on three different days. Diabetes was defined by meeting any of the following criteria (Genuth et al., 2018): (a) a previous diagnosis of diabetes by a healthcare professional or current use oral antidiabetic medications or insulin; (b) fasting blood glucose (FBG) ≥ 7.0 mmol/L or glycated hemoglobin (HbA1C) ≥ 6.5%.

### 2.4 Determination of PD

The diagnosis of PD was based on the prescription of one or more medications specifically PD used for its treatment. In the NHANES database, the use of certain medications is assessed through survey responses. Due to limitations in data collection, not all medication used to treat PD can be included. Consequently, the NHANES database, includes only a subset of these medications, such as Carbidopa, Entacapone, Levodopa, Amantadine, Pramipexole, and Bromocriptine (Ke et al., 2024; Zhou X. et al., 2024). Individuals were classified as having PD only if they report using one or more of these specified medications; otherwise, they were categorized as non-PD participants.

### 2.5 Follow-up and outcome assessment

PD prevalence and all-cause mortality were the primary outcome measures. The participants’ vital status was verified by consulting the National Death Index (NDI).^2^ In the NHANES database, follow-up data were recorded monthly. The follow-up information included survival status at the time of follow-up, survival time, cause of death, and other relevant details. Causes of death were categorized into several broad categories, including: traffic accidents, heart disease, cerebrovascular accidents, pneumonia and influenza, nephritis, kidney disease and nephrotic syndrome, malignant neoplasms, chronic respiratory diseases, diabetes mellitus, and other causes. All-cause mortality referred to death from any cause that occurred during the follow-up period.

### 2.6 Statistical analysis

Data analysis was performed using R (version 4.1.1), adhering to the NHANES database’s complex, multi-stage, and multi-stratified sampling design by incorporating the recommended weighting variables from the data provider. The “gtsummary” package in R was employed for weighted statistical analysis. Continuous variables were initially tested for normality, which revealed deviations from a normal distribution. Consequently, non-normally distributed variables were reported using the median and the 25th and 75th percentiles, and statistical comparisons were performed using the Wilcoxon test. Categorical variables were expressed as frequencies and proportions, with statistical analyses performed using the chi-square test.

To analyze the relationship between CDAI exposure and the prevalence of PD, we employed weighted logistic regression and constructed three models by adjusting for various variables. The odds ratio (OR) was used to determine whether CDAI exposure is a risk factor, with OR < 1 indicating a protective factor and OR > 1 indicating a risk factor. After establishing the relationship between CDAI exposure and PD prevalence, we selected the PD cohort and further investigated the association between CDAI exposure and all-cause mortality using a weighted multivariate Cox regression model. The hazard ratio (HR) was utilized to assess the relationship between CDAI exposure and mortality outcomes, with HR < 1 indicating a protective factor and HR > 1 indicating a risk factor. In the multivariate weighted Cox regression analysis, three models were applied to account for potential confounding effects. Subsequently, subgroup analyses were conducted to examine whether the relationship between CDAI exposure and all-cause mortality persists across different subgroups within the PD population, such as those aged over 60, those with hypertension or diabetes, and individuals who smoke or consume alcohol. Interaction analyses were performed to determine if CDAI acts as an independent influencing factor.

Finally, we performed a quantification analysis of CDAI exposure and outcome events using restricted cubic spline (RCS) analysis. RCS curves were used to identify specific levels of CDAI exposure associated with adverse effects on the outcome events. This approach facilitates a detailed understanding of how varying levels of CDAI exposure influence the risk of adverse outcomes. In statistical analysis, a *p* < 0.05 is generally considered indicative a statistically significant difference.

## 3 Results

### 3.1 Characteristics of the study population

This cohort study initially included 34,133 participants, of whom 510 were diagnosed with PD, representing a weighted proportion of 1.4%. Among the participants, 16,054 were male, accounting for a weighted proportion of 45%. Based on the screening criteria for PD, participants were categorized into PD and non-PD groups. Statistically significant differences were observed between the two groups in terms of age, gender, education level, family poverty index (PIR), and smoking status. Additionally, laboratory tests revealed statistically significant differences between the two groups in blood creatinine, albumin (ALB), alanine aminotransferase (ALT), total bilirubin, triglycerides, total cholesterol, and fasting blood glucose levels.

Compared to individuals without PD, those with PD tended to be older, had lower family income levels, and lower educational attainment, but are more likely to engage in smoking. In contrast, individuals without PD tend to have lower levels of blood creatinine, triglycerides, total cholesterol, and fasting blood glucose. Interestingly, CDAI levels were significantly higher in the non-PD group compared to the PD group, suggesting that CDAI might serve as a protective factor (Table 1).

**Table 1:** Basic demographic information of the research subjects.

Characteristic	*N* ^1^	Overall *N* = 34,133 (100%)^2^	Non-PD *N* = 33,623 (99%)^2^	PD *N* = 510 (1.4%)^2^	*P*-value^3^
Age (Years)	34,133	57 (43, 68)	57 (43, 68)	59 (47, 75)	<0.001**
Gender	34,133				0.015*
Female		18,079 (55%)	17,787 (55%)	292 (62%)	
Male		16,054 (45%)	15,836 (45%)	218 (38%)	
Race	34,133				0.700
Mexican American		3,910 (6%)	3,873 (6%)	37 (5%)	
Other Hispanic		3,139 (4%)	3,096 (4%)	43 (6%)	
Non-Hispanic White		17,036 (73%)	16,707 (73%)	329 (74%)	
Non-Hispanic Black		6,864 (10%)	6,798 (10%)	66 (9%)	
Other race		3,184 (7%)	3,149 (7%)	35 (6%)	
PIR	34,133	2.73 (1.41, 4.88)	2.75 (1.41, 4.92)	2.03 (1.11, 3.09)	<0.001**
Education	34,133				<0.001**
<High School		8,998 (17%)	8,850 (17%)	148 (25%)	
High School or some College		18,110 (55%)	17,837 (55%)	273 (55%)	
College or above		7,025 (27%)	6,936 (27%)	89 (20%)	
Smoking	34,133				0.031*
No		16,766 (50%)	16,527 (50%)	239 (44%)	
Yes		17,367 (50%)	17,096 (50%)	271 (56%)	
Drinking	34,133				0.200
No		10,205 (26%)	10,038 (26%)	167 (30%)	
Yes		23,928 (74%)	23,585 (74%)	343 (70%)	
Hypertension	34,133				0.400
No		12,604 (43%)	12,408 (43%)	196 (40%)	
Yes		21,529 (57%)	21,215 (57%)	314 (60%)	
Diabetes	34,133				0.054
No		21,952 (72%)	21,579 (72%)	373 (77%)	
Yes		12,181 (28%)	12,044 (28%)	137 (23%)	
BMI (kg/m^2^)	34,133	29.20 (25.20, 34.17)	29.20 (25.20, 34.20)	29.51 (24.40, 33.50)	0.400
Creatinine (mg/dL)	34,133	0.86 (0.73, 1.02)	0.86 (0.73, 1.02)	0.88 (0.76, 1.05)	0.016*
Albumin (g/L)	34,133	42.00 (40.00, 44.00)	42.00 (40.00, 44.00)	41.00 (39.00, 43.00)	0.002**
ALT(U/L)	34,133	21.00 (16.00, 28.00)	21.00 (16.00, 28.00)	19.00 (14.00, 25.00)	<0.001**
AST(U/L)	34,133	23.00 (19.00, 27.00)	23.00 (19.00, 27.00)	23.00 (19.00, 27.00)	0.900
Total bilirubin (umol/L)	34,133	10.26 (8.55, 13.68)	10.26 (8.55, 13.68)	8.55 (6.84, 11.97)	<0.001**
Triglycerides (mmol/L)	34,133	1.24 (0.86, 1.81)	1.23 (0.86, 1.81)	1.51 (1.30, 1.51)	<0.001**
Total cholesterol (mmol/L)	34,133	4.76 (4.09, 5.48)	4.76 (4.09, 5.48)	4.82 (4.27, 5.72)	0.009**
Glycosylated hemoglobin (%)	34,133	5.60 (5.30, 6.10)	5.60 (5.30, 6.10)	5.60 (5.30, 6.10)	>0.900
Fasting blood glucose (mmol/L)	34,133	5.77 (5.27, 6.55)	5.77 (5.27, 6.55)	6.62 (5.72, 6.62)	<0.001**
Systolic pressure (mmHg)	34,133	123 (113, 135)	123 (113, 135)	122 (110, 135)	0.800
Diastolic pressure (mmHg)	34,133	69 (62, 77)	69 (62, 77)	69 (63, 77)	0.300
Vitamin A (mcg)	34,133	−0.04 (−0.45, 0.53)	−0.04 (−0.45, 0.53)	−0.04 (−0.54, 0.50)	0.300
Vitamin C (mg)	34,133	−0.17 (−0.44, 0.26)	−0.17 (−0.44, 0.26)	−0.18 (−0.49, 0.30)	0.500
Vitamin E (mg)	34,133	0.06 (−0.39, 0.63)	0.06 (−0.39, 0.63)	−0.12 (−0.51, 0.49)	<0.001**
Selenium (mcg)	34,133	0.02 (−0.28, 0.52)	0.02 (−0.28, 0.52)	−0.04 (−0.45, 0.33)	<0.001**
Zinc (mg)	34,133	0.18 (−0.28, 0.69)	0.18 (−0.28, 0.69)	0.03 (−0.44, 0.61)	0.010*
Carotenoids (mcg)	34,133	−0.17 (−0.52, 0.51)	−0.17 (−0.52, 0.51)	−0.32 (−0.62, 0.39)	0.002**
CDAI	34,133	0.72 (−1.29, 3.17)	0.73 (−1.28, 3.18)	0.09 (−1.87, 2.71)	0.012*

^1^N not Missing (unweighted). ^2^Median (IQR) for continuous; *n* (%) for categorical. ^3^Wilcoxon rank-sum test for complex survey samples; chi-squared test with Rao and Scott’s second-order correction. PIR, Ratio of family income to poverty; BMI, Body mass index; ALT, Alanine aminotransferase; AST, Aspartate aminotransferase; CDAI, Composite Dietary Antioxidant Index; PD, Suffering from Parkinson’s disease; Non-PD, Not suffering from Parkinson’s disease. *p < 0.05, **p < 0.01. *p* < 0.05 was considered statistically significant.

### 3.2 CDAI exposure as an independent protective factor for PD risk

To clarify the association between CDAI exposure and the prevalence of PD, we conducted multivariate weighted logistic regression analysis to calculate the odds ratios (OR) and their 95% confidence intervals (Table 2). In the logistic regression analysis, the continuous CDAI variable was divided into three groups based on its tertiles: Q1 (<−1.07), Q2 (−1.07 to 1.74), Q3 (>1.74).

**Table 2:** Multivariate weighted logistic regression analysis of the association between CDAI exposure and the prevalence of PD.

Model	CDAI group	OR^1^	95% CI^1^	*p*-value
Model 1				0.027*
	Q1	–	–	
	Q2	0.68	0.50, 0.94	0.020*
	Q3	0.69	0.51, 0.93	0.015*
Model 2				0.047*
	Q1	–	–	
	Q2	0.69	0.50, 0.95	0.035*
	Q3	0.72	0.54, 0.98	0.025*
Model 3				0.049*
	Q1	–	–	
	Q2	0.70	0.51, 0.96	0.027*
	Q3	0.72	0.54, 0.98	0.035*

^1^OR, Odds Ratio; CI, Confidence Interval. Model 1: No confounding factors were adjusted. Model 2: Adjusted for Age, Gender, Hypertension, Diabetes. Model 3: Adjusted for Age, Gender, Hypertension, Diabetes, Fasting blood glucose levels, ALT, Creatine, Triglyceride and Total cholesterol. *p < 0.05, *p* < 0.05 was considered statistically significant.

In Model 1, no adjustments were made for confounding factors. In Model 2, we adjusted for age, gender, hypertension, and diabetes status. Model 3 included additional adjustments for Fasting blood glucose levels, ALT, Creatine, Triglyceride and total cholesterol. Multivariate weighted logistic regression analysis indicated that higher levels of CDAI exposure were associated with a reduced risk of developing PD. Compared to the first tertile of CDAI levels, the odds ratio for PD prevalence in the third tertiles (Q3 vs. Q1) was reduced by approximately 31%. After adjusting for various confounding variables, this relationship remained significant in both Models 2 and 3.

### 3.3 Negative correlation between CDAI exposure levels and all-cause mortality in PD patients

To determine whether CDAI continues to serve as a protective factor for individuals already diagnosed with PD, we conducted a survival analysis using a weighted Cox regression model. Similar to the logistic regression analysis, continuous CDAI values were categorized into groups based on their tertiles for the Cox regression analysis (Table 3).

**Table 3:** Multivariate weighted cox regression analysis of CDAI and All-cause mortality in PD patients.

Model	CDAI group	HR^1^	95% CI^1^	*p*-value
Model 4				0.018*
	Q1	—	—	
	Q2	0.56	0.37, 0.85	0.007**
	Q3	0.64	0.38, 1.07	0.088
Model 5				0.025*
	Q1	—	—	
	Q2	0.58	0.35, 0.93	0.047*
	Q3	0.54	0.32, 0.90	0.025*
Model 6				0.017*
	Q1	—	—	
	Q2	0.56	0.35, 0.89	0.015*
	Q3	0.53	0.31, 0.90	0.018*

^1^HR, Hazard Ratio; CI, Confidence Interval. Model 4: no confounding factors were adjusted. Model 5: adjusted for Age, Gender, Hypertension, Diabetes. Model 6: adjusted for Age, Gender, Hypertension, Diabetes, Fasting blood glucose levels, ALT, BMI, Triglyceride and Total cholesterol. *p < 0.05, **p < 0.01. *p* < 0.05 was considered statistically significant.

In Model 4, no adjustments were made for any variables. Model 5 included adjustments for age, gender, hypertension, and diabetes status. In Model 6, further adjustments were made for fasting blood glucose, triglycerides, total cholesterol, BMI, and ALT. In individuals already diagnosed with PD, CDAI levels were negatively correlated with all-cause mortality, indicating that higher CDAI levels are associated with a reduced risk of all-cause mortality in this population. This conclusion is supported by the following fact: In Table 3, the hazard ratio (HR) for all-cause mortality in the third tertile of CDAI is approximately 36% lower compared to the first tertile (Q3 vs. Q1). In Model 6, the HR was even 47% lower in the third tertile of CDAI compared to the first tertile, further indicating that higher levels of CDAI exposure may protect PD patients from all-cause mortality.

### 3.4 Subgroup analysis and interaction analysis

**Figure 2:**
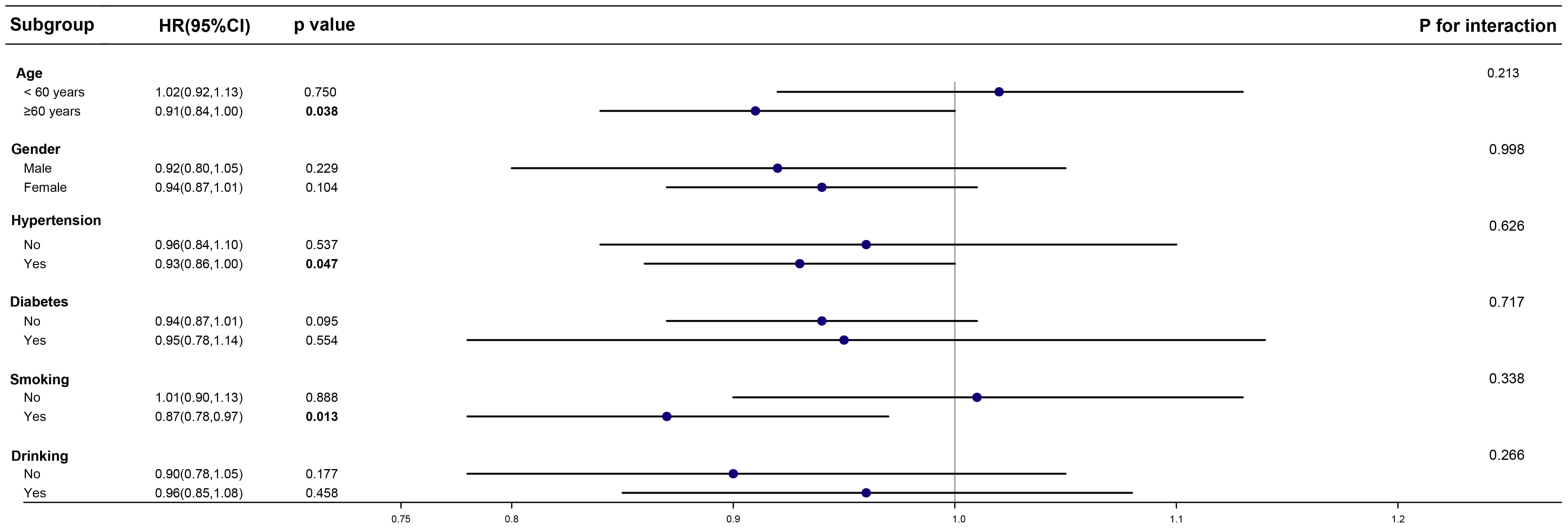
Effect size of CDAI on the presence of PD in the age, gender, hypertension, diabetes and drinking subgroup.

After establishing CDAI as a protective factor, we sought to determine whether this protective effect persists across different subgroups and whether it remains an independent factor. To address these questions, we conducted subgroup and interaction analyses (Figure 2).

In the PD cohort, we categorized individuals into two groups based on age (age ≥ 60 years and age < 60 years). We also included subgroups based on gender, hypertension status, diabetes status, smoking status, and alcohol consumption. The results of the subgroup analysis indicated that in individuals already diagnosed with PD, the protective effect of CDAI is more pronounced in those aged over 60, or those with hypertension or smoke. However, no significant differences were found between CDAI exposure and all-cause mortality in the subgroups with diabetes, alcohol consumption, or across different genders. This lack of distinction may be due to confounding factors affecting these subgroups, which could have introduced errors into the analysis. The interaction analysis showed that CDAI exposure was an independent factor influencing all-cause mortality, unaffected by age, gender, the presence of underlying conditions, or tobacco and alcohol use.

### 3.5 Quantitative analysis of CDAI exposure levels and outcomes: restricted cubic spline regression analysis

**Figure 3:**
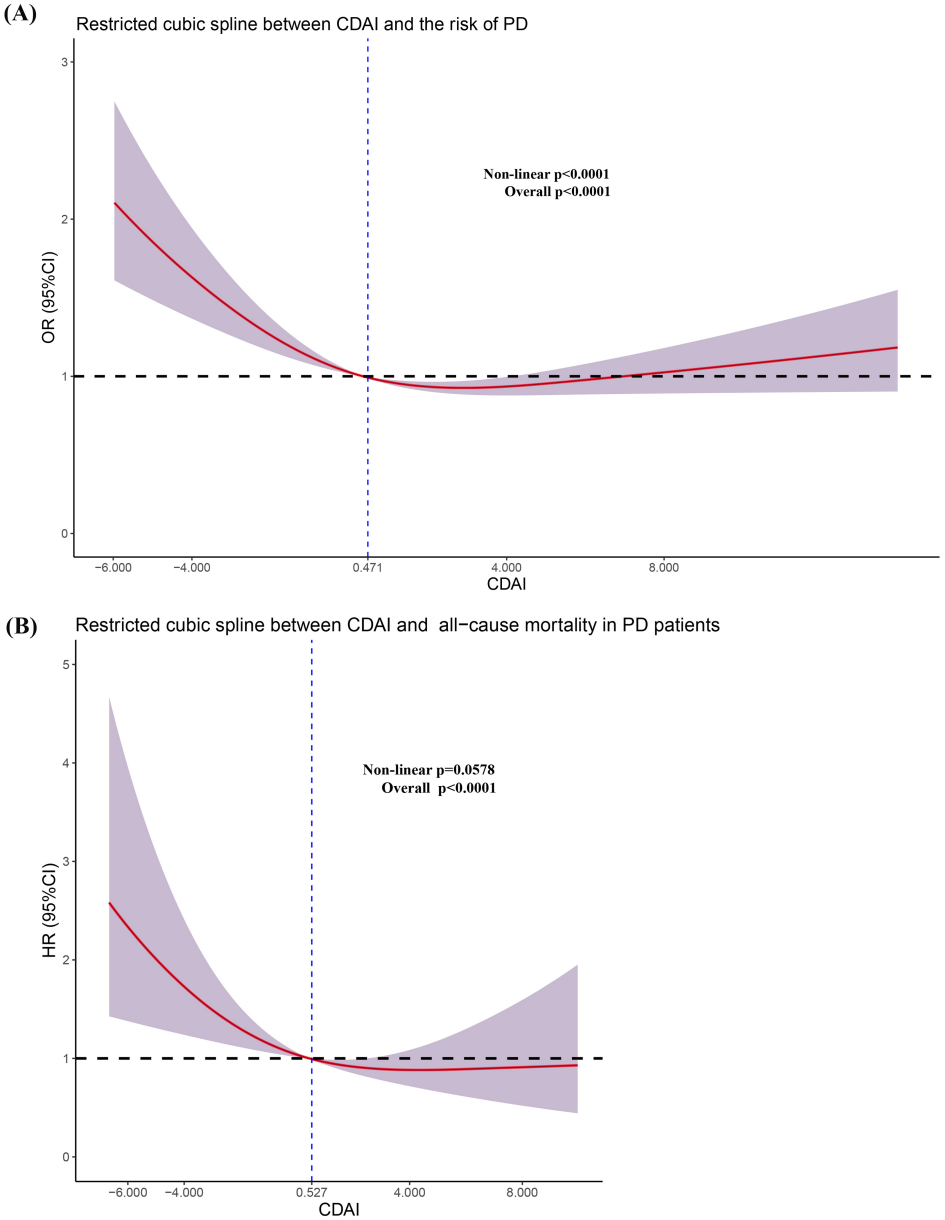
Restricted cubic spline regression analysis between CDAI and the risk of PD **(A)** and all-cause mortality in PD patients **(B)**.

Finally, we conducted a restricted cubic spline regression analysis to identify the specific levels of CDAI exposure levels associated with adverse outcomes (Figure 3).

First, we performed a nonlinear test to clarify the quantitative relationship between CDAI and the odds ratios (OR) or hazard ratios (HR). We found that CDAI exhibited a nonlinear relationship (non-linear *p* < 0.0001) with odds ratios (OR), but a linear relationship (non-linear *p* = 0.0578) with hazard ratios (HR). In Figure 3A, the odds ratio (OR) equals 1 at a CDAI value of 0.471. For CDAI values below 0.471, the OR decreases nonlinearly as CDAI levels rise, demonstrating a nonlinear reduction in the odds of the outcome with increasing CDAI levels. However, when CDAI exceeded 0.471, the OR initially decreased with increasing CDAI levels, but subsequently begins to rise, eventually surpassing 1. As CDAI continues to increase, the 95% CI for OR spans both values >1 and <1, indicating a lack of statistical significance. Therefore, we concluded that CDAI values <0.471 represent an independent risk factor for PD, while increases in CDAI within a certain range reduce the risk of developing PD.

Similar findings were observed in the Cox regression analysis. Specifically, we found that CDAI values below 0.527 are associated with an increased risk of all-cause mortality in the PD population. The quantitative analysis results indicated that maintaining CDAI levels above a certain threshold is beneficial for reducing the prevalence of PD and all-cause mortality. This suggests that increasing the intake of antioxidant-rich elements through diet or supplements could benefit the population.

## 4 Discussion

In this cross-sectional study, using data from the 2007–2018 NHANES involving 34,133 US participants, we found that participants in the middle and highest CDAI score tertiles exhibited a significantly lower risk (approximately 30%) of developing PD compared to those in the lowest CDAI tertile, even after adjusting for multiple confounders. Additionally, we observed a nonlinear relationship between CDAI and PD prevalence, with a calculated turning point at 0.471. Furthermore, within the PD cohort, CDAI remains a protective factor. Specifically, a CDAI value below 0.527 significantly heightens the risk of all-cause mortality. This effect is particularly pronounced among individuals over the age of 60, smokers, or those with comorbid hypertension. These findings suggest that lower CDAI level is an independent risk factor for both PD development in the general population and all-cause mortality in PD patients (cutoffs: <0.471 for PD risk; <0.527 for mortality).

Oxidative stress is a pivotal factor in the pathogenesis of PD (Imbriani et al., 2022). In the context of PD, there is a marked increase in reactive oxygen species (ROS) and reactive nitrogen species (RNS) levels (Trist et al., 2019). These free radicals exert detrimental effects by targeting cellular components, including lipids, proteins, and DNA, which leads to cellular damage and apoptosis (Raza et al., 2019; Dong-Chen et al., 2023). Of particular significance is the impact of oxidative stress on dopaminergic neurons, where it contributes substantially to the progression of the disease. The resultant oxidative damage to these critical neurons is a central element driving the advancement of PD, highlighting the importance of oxidative stress in the disease’s etiology and progression (Subramaniam and Chesselet, 2013; Cerri et al., 2019; Dong-Chen et al., 2023). Oxidative stress results in mitochondrial dysfunction, a critical pathological alteration in PD (Bose and Beal, 2016). Mitochondria, which function as the cellular energy generators, are also a primary source of free radical production. In PD, mitochondrial impairment leads to reduced ATP synthesis and exacerbates oxidative stress, further compromising dopaminergic neurons (Dauer and Przedborski, 2003; Subramaniam and Chesselet, 2013; Raza et al., 2019; Moradi Vastegani et al., 2023). The elevated production of free radicals instigates neuroinflammatory responses, with inflammatory mediators perpetuating oxidative stress and establishing a deleterious feedback loop (Rocha et al., 2018; Jankovic and Tan, 2020). This cyclical process significantly contributes to neuronal damage and eventual neurodegeneration. Moreover, in the context of PD, the functionality of the cellular antioxidant defense system, including antioxidant enzymes and scavengers, may be severely compromised (Hatziagapiou et al., 2019; Tahavvori et al., 2023). This attenuation of antioxidant capacity exacerbates oxidative stress, thereby intensifying the progression of the disease (Tchekalarova and Tzoneva, 2023).

Emerging evidence underscores a robust association between dietary patterns and PD risk and progression (Xu et al., 2023). Diets rich in antioxidants, such as the Mediterranean diet, are linked to reduced PD risk, whereas pro-inflammatory diets high in processed foods may exacerbate neurodegeneration. Diet also profoundly shapes gut microbial communities, which are increasingly recognized as mediators of PD pathogenesis (Sobral et al., 2025). PD patients exhibit distinct gut dysbiosis (reduced Prevotellaceae and increased Bifidobacterium), compared to healthy controls (Aho et al., 2019; Sobral et al., 2025). This gut-brain axis, a bidirectional conduit linking intestinal health to neurodegeneration, is mechanistically implicated in PD pathology: microbial products may trigger *α*-synuclein aggregates in the enteric nervous system, which propagate via the vagus nerve to the substantia nigra(Rietdijk et al., 2017), while gut-derived lipopolysaccharides (LPS) and inflammatory cytokines activate microglia, driving neuroinflammation and neuronal death(Sampson et al., 2016). These alterations correlate with gastrointestinal symptoms and α-synuclein aggregation, a hallmark of PD pathology. Collectively, these findings suggest that diet likely influences PD through synergistic mechanisms, including antioxidant-mediated neutralization of reactive oxygen species (ROS) to protect nigrostriatal neurons and gut-brain axis modulation via dietary reshaping of microbiota, which reduces endotoxin translocation and inflammatory signaling to the brain.

The CDAI has increasingly become a central focus in the field of research concerning dietary antioxidants. Its prominence reflects growing interest in understanding how comprehensive dietary patterns impact oxidative stress and overall health. Diet plays a pivotal role in modulating the body’s oxidative stress levels (Aleksandrova et al., 2021; Młynarczyk et al., 2022). Vitamin E and selenium, as critical antioxidant vitamins, possess the ability to effectively scavenge and neutralize free radicals, thereby mitigating the severity of oxidative stress (Calik et al., 2022; Jung et al., 2023; Mehvari et al., 2023; Mesalam et al., 2023). Our study revealed that levels of vitamin E and selenium were significantly lower in individuals with PD compared to the non-PD group (Table 1). Furthermore, the levels of zinc and carotenoids were significantly lower in the PD group compared to the non-PD group. Existing research underscores that zinc and carotenoids are also crucial antioxidants with significant roles in oxidative stress mitigation (Riccioni, 2009; Roehrs et al., 2017; Genç et al., 2020; Aazami et al., 2021). Although our analysis revealed no statistically significant differences in dietary intake levels of vitamin A and vitamin C between the PD group and the non-PD group, it is crucial to note that CDAI is a composite measure. In this context, reductions were observed in four other components (vitamin E, zinc, selenium, and carotenoids) within the PD cohort. This suggests that these four nutrients may play a more critical role in the prophylaxis of PD. Collectively, the markedly lower CDAI values in the PD group, compared to the non-PD group, reflect a pronounced diminishment in antioxidant defense mechanisms. This finding elucidates why a reduction in CDAI correlates with an increased risk of developing PD.

Furthermore, among individuals already afflicted with PD, a sustained decline in CDAI exacerbates the risk of all-cause mortality, especially among individuals over the age of 60, smokers, or those with comorbid hypertension. Aging itself is marked by progressive oxidative stress accumulation and declining endogenous antioxidant defenses, with a notable acceleration in biological aging processes observed around the sixth decade of life (Shen et al., 2024). Concurrently, smoking induces chronic oxidative stress via reactive oxygen species (ROS) from tobacco combustion and inflammation, while hypertension promotes endothelial dysfunction and vascular oxidative damage via angiotensin II-induced NADPH oxidase activation and ROS overproduction (Qin et al., 2024; Zhao et al., 2025). Importantly, higher CDAI levels in hypertensive populations have been linked to reduced risks of all-cause mortality, cardiovascular mortality, and cancer-related mortality (Qin et al., 2024; Zhao et al., 2025). In PD patients with hypertension, this vascular oxidative burden synergizes with neuroinflammation, further amplifying neuronal oxidative damage. Consequently, older adults, smokers, or hypertensive individuals with PD likely experience synergistic oxidative insults, making them more dependent on exogenous antioxidants from diet. A suboptimal CDAI fails to counteract this dual pathogenic burden, thereby accelerating cellular degeneration and mortality risk in these vulnerable subgroups. In the Restricted cubic spline regression analysis (Figure 3), we observed that when CDAI <0.471, the OR was >1, and the entire curve along with its 95% confidence interval (CI) was situated above the value of 1. However, as CDAI continued to increase, the 95% CI of the curve spanned both above and below 1, indicating that this portion of the curve may lack statistical significance. Therefore, we can only conclude that CDAI values <0.471serves as an independent risk factor for the occurrence of PD in the population. Research has consistently demonstrated the protective effects of the CDAI against both all-cause and cardiovascular mortality (Sheng et al., 2022; Tan et al., 2023; Luo J. et al., 2024; Peng and Fang, 2024; Zhang et al., 2024). Nevertheless, in the context of PD, the incidence of cardiovascular mortality is relatively rare, suggesting that cardiovascular death may not be a predominant cause of mortality within this cohort. To mitigate the risk of substantial bias and inaccuracies in statistical analysis due to the sparse occurrence of cardiovascular mortality events, we refrained from including an analysis of cardiovascular mortality risk in our study. Therefore, it is imperative that future, more extensive and methodologically rigorous cohort studies are conducted to elucidate the potential relationship between CDAI and cardiovascular mortality risk in PD patients.

This study carries significant implications, as our multivariate-adjusted analysis (accounting for confounders such as age and gender) demonstrates that the CDAI effectively predicts both PD risk (OR = 0.72) and mortality (HR = 0.53), suggesting its utility as a stratification tool for identifying high-risk subgroups. The established CDAI thresholds (<0.471 for PD risk; <0.527 for mortality) enable targeted personalized nutritional interventions, underscoring the need for healthcare providers to prioritize antioxidant-rich dietary recommendations in individuals below these critical values. Furthermore, integrating CDAI with digital biomarkers (e.g., gait analytics) and *α*-synuclein-related metabolomic profiles could enhance prognostic precision, aligning with evidence that multimodal data integration improves PD comorbidity prediction(Brzenczek et al., 2024). However, several limitations should be acknowledged in this study. First, the observational design and retrospective nature of the NHANES data preclude causal inference, limiting our findings to associative relationships that may be influenced by residual confounding from unmeasured variables (e.g., genetic predisposition, environmental exposures). Second, reliance on self-reported dietary recall introduces inherent risks of measurement error, including recall bias and misclassification of nutrient intake, which may compromise CDAI calculations. The absence of longitudinal dietary monitoring further compounds these limitations, as static assessments fail to capture temporal variations in dietary patterns that could affect outcome associations. Third, while the NHANES cohort provides nationally representative U.S. data, the generalizability of our results to global populations remains uncertain due to marked cross-cultural differences in dietary practices (e.g., Mediterranean vs. Asian dietary patterns) and genetic susceptibility profiles. Future multinational cohorts incorporating culturally adapted CDAI thresholds and dynamic dietary tracking are needed to validate these findings across diverse populations.

## 5 Conclusion

Our cross-sectional study reveals that reduced CDAI levels, especially when falling below the threshold of 0.471, as a significant independent risk factor for PD development. Moreover, among patients with established PD, a CDAI level below 0.527 significantly heightens the risk of all-cause mortality, particularly in older adults (>60 years), active smokers, and hypertensive individuals. These findings highlight the critical role of dietary antioxidant capacity in PD pathophysiology and prognosis. We propose that targeted personalized dietary interventions rich in antioxidants or antioxidant supplementation may represent a novel preventive/therapeutic strategy for high-risk populations with suboptimal CDAI level and PD patients.

## Data Availability

The original contributions presented in the study are included in the article/supplementary material, further inquiries can be directed to the corresponding author.

## Footnotes

^1^ https://www.cdc.gov/nchs/nhanes/

^2^ https://ftp.cdc.gov/pub/Health_Statistics/NCHS/datalinkage/linked_mortality/
